# Is lack of goal-conflict-specific rhythmicity a biomarker for treatment resistance in generalised anxiety but not social anxiety or major depression?

**DOI:** 10.1177/02698811241275627

**Published:** 2024-09-02

**Authors:** Shabah M Shadli, Carina J Donegan, Muhammad SS Bin Mohd Fahmi, Bruce R Russell, Paul Glue, Neil McNaughton

**Affiliations:** 1 Department of Psychology, University of Otago, Dunedin, New Zealand; 2 School of Psychology, Charles Sturt University, Bathurst, NSW, Australia; 3 School of Psychology, University of Auckland, Auckland, New Zealand; 4 School of Pharmacy, University of Otago, Dunedin, New Zealand; 5 Department of Psychological Medicine, University of Otago, Dunedin, New Zealand

**Keywords:** Anxiety, depression, treatment resistance, anxiety biomarker, EEG

## Abstract

**Background:**

Anxiety and depression cause major detriment to the patient, family, and society – particularly in treatment-resistant (TR) cases, which are highly prevalent. TR prevalence may be due to current diagnoses being based not on biological measures but on symptom lists that suffer from clinical subjectivity, variation in symptom presentation, and comorbidity.

**Aims:**

Goal-conflict-specific rhythmicity (GCSR) measured using the Stop-Signal Task (SST) may provide the first neural biomarker for an anxiety process and disorder. This GCSR has been validated with selective drugs for anxiety. So, we proposed that GCSR could differ between TR and non-TR individuals and do so differently between those diagnoses normally sensitive to selective anxiolytics and those not.

**Methods:**

We recorded electroencephalograms (EEG) from 20 TR participants (4 GAD, 5 SAD and 11 MDD) and 24 non-TR participants (4 GAD, 5 SAD and 15 Comorbid GAD/MDD (GMD)) while they performed the SST.

**Results:**

There was significant positive GCSR in all groups except the GAD-TR group. GAD-TR lacked GCSR in the low-frequency range. However, TR had little effect in SAD or MDD/GMD populations with apparent increases not decreases.

**Conclusions:**

Overall, these results suggest that GAD may occur in two forms: one resulting from excessive GCSR and so being drug sensitive, and the other resulting from some other mechanism and so being TR. In SAD and MDD groups, heightened GCSR could be a consequence rather than the cause, driven by mechanisms that are normally more sensitive to non-selective panicolytic antidepressants.

## Introduction

‘Neurotic disorders’ such as anxiety and depression share high neuroticism as both a risk and a maintaining factor (McNaughton and Glue, 2020). These disorders are a leading cause of health disability globally (Brakowski et al., 2017; George et al., 2017; Kessler, 2003; Mulders et al., 2015). They have high chronicity, severity, costs to public health (Oakley-Browne et al., 2006), and suicide risk (Roy-Byrne et al., 2000). Worse, around one-third of anxious or depressed individuals are treatment resistant (TR; Bystritsky, 2006; Souery et al., 2006) – generally defined as a lack of improvement with conventional treatments, usually, after at least two drugs and psychotherapy have been tested (Bokma et al., 2019; Roy-Byrne, 2015). Comorbid anxiety and depression have an even higher percentage of TR individuals than depression or anxiety alone (Coplan et al., 2015; Souery et al., 2007).

TR prevalence may be due to symptom-based diagnostic frameworks and the differences between them (Vermani et al., 2011). Current diagnoses are based on symptom lists and duration requirements that suffer from clinical subjectivity, comorbidity, and variation in symptom presentation. This is akin to diagnosing COVID-19 from a cough and high temperature rather than the presence of the SARS-CoV-2 virus (McNaughton, 2018). Instead, the hope is ‘that identifying syndromes based on pathophysiology will eventually be able to improve outcomes’ (Insel et al., 2010: 748).

Goal-conflict-specific rhythmicity (GCSR) measured using the Stop-Signal Task (SST) may help solve these problems by providing the first neural biomarker for an anxiety process. According to *The Neuropsychology of Anxiety* (Gray and McNaughton, 2000; McNaughton and Gray, 2024), goal-conflict detection and resolution is a key property of the neural systems that mediate the action of selective drugs for anxiety (i.e. those that are not also panicolytic). In the SST, there is a ‘horse-race’ between parallel going/approach and stopping/avoidance processes (Logan et al., 1984) so variation in the delay of the stop signal (SSD) can produce approach, avoidance or a conflict between them. To extract conflict-specific activity in the SST, EEG power from each participant is first averaged across approach (long SSD with going predominating) and avoidance (short SSD with stopping predominating) trials where conflict is expected to be low. This average cancels out the effects of factors related simply to time in the trial and to any progressive changes in go-and-stop processes themselves with SSD change. This average approach + avoidance power is then subtracted from intermediate SSD power, that is, approach-avoidance periods (Neo et al., 2011) to generate conflict-specific power. This occurs at right frontal (F8) scalp sites in the 4–10 Hz band (usually peaking at 8 Hz) in right-handed individuals (McNaughton et al., 2013; Neo and McNaughton, 2011; Shadli et al., 2015), consistent with SST control by the right inferior frontal gyrus (Aron et al., 2003).

As expected, this GCSR is positively related to Spielberger’s State Trait Anxiety Inventory – Trait scale (STAI-T; Spielberger et al., 1983) and is reduced by single doses of selective drugs for anxiety, that is, buspirone, triazolam or pregabalin (McNaughton et al., 2013; Shadli et al., 2015), which act as a receptor partial agonist (5-HT1A), a Positive Allosteric Modulator (GABA-A receptor, benzodiazepine site), and a voltage-gated calcium channel blocker, respectively. Thus, GCSR in humans measured during the SST represents a biomarker for goal-conflict system activation and thus one key anxiety process (Gray and McNaughton, 2000; McNaughton and Corr, 2004; McNaughton and Gray, 2024).

Shadli et al. (2021a) demonstrated that, in contrast to similar STAI-T scores across clinical anxiety diagnoses, GCSR amplitude and frequency spectrum shape varied. In particular, maximum power in the 4–7 Hz range, when averaged across participants, varied with diagnosis. The average values ranked control < generalised anxiety < comorbid generalised anxiety and depression < mixed other diagnoses < social anxiety. Such variation (but not with the same ordering) can be seen both within and between biotypes defined by different patterns of connectivity within the default mode, salience and attention circuits as assessed by fMRI (Tozzi et al., 2024) – with the biotypes varying in their response to different therapies.

Notably, higher GCSR appears to occur more often in diagnoses that tend to be less sensitive to selective drugs for anxiety. In particular, individuals with comorbid generalised anxiety and depression (GMD) had a higher 4–7 Hz average than individuals with GAD but without comorbid depression. These GMD individuals are also more likely to be TR than individuals diagnosed with GAD or MDD alone (Coplan et al., 2015; Souery et al., 2007). Thus, differences in GCSR (i.e. being higher in a population with a larger TR prevalence) may allow us to predict individuals that will be TR.

The present study recruited TR participants with GAD, SAD and MDD and compared them to non-TR cases drawn from a previous pool. GAD and SAD were demographically matched by selection from the large available non-TR pool. TR-MDD were compared, without individual matching to our entire set of non-TR GMD as this non-TR pool lacked pure MDD cases and there were insufficient GMD for demographic matching. We measured GCSR in the SST and we then compared TR and non-TR cases across the three diagnoses.

*Hypothesis 1*: Both TR and non-TR anxiety cases will demonstrate a clear GCSR response as with previous anxiety disorder patients and in contrast to previous low-STAI-T healthy controls (see Shadli et al., 2021a).
*Hypothesis 2*: TR individuals with anxiety will have a larger GCSR than treatment-responsive individuals independent of GAD or SAD diagnoses with SAD cases of both types having larger GCSR than GAD cases (see Shadli et al., 2021a).
*Hypothesis 3*: TR will have a larger positive effect on GCSR in SAD than in GAD patients.
*Hypothesis 4*: TR-MDD (if representing a pure depression diagnosis) will have less strong GCSR than GMD (given that GMD has comorbid anxiety and has previously shown substantial GCSR; Shadli et al., 2021a).

## Methods

### Participants

There were 44 participants (20 TR and 24 non-TR). Ten participants (5 TR/5 non-TR) were diagnosed with SAD, eight (4 TR/4 non-TR) with GAD, 11 with MDD (TR) and 15 with GAD/comorbid MDD (GMD; non-TR).

TR participants were recruited on entry to a separate in-progress project analysing ketamine’s therapeutic effects. Our criterion for TR as stated in our approved ethics protocol was that patients ‘have not responded to at least two adequate trials of relevant medication and at least one trial of relevant psychotherapy’. Testing reported here was carried out before ketamine treatment.

GAD-non-TR and SAD-non-TR participants were selected using demographic matching criteria from a data set published by Shadli et al. (2021a). Non-TR-GMD were selected from the same published data set but were too few to match demographically. Participants were aged between 20 and 49 (*M* = 29). Thirty-two were females and 12 were males. Participants were right-handed, except for one MDD and two GMD patients – all were included in the analysis. Participant diagnoses were obtained using the Mini International Neuropsychiatric Interview (ver 6.0; American Psychiatric Association, 1994). Participants were given a numerical ID to which their demographic, behavioural and EEG data were assigned. This was kept separate from any identity information.

The study received ethical approval from the New Zealand Health and Disability Ethics Committees (9/CEN/21/AM03).

### Materials

Participants supplied demographic information (age, weight, handedness, gender, ethnic group: standard statistics NZ format) and then answered two sets of questionnaires included as part of a larger personality-related study.

Set 1, before the SST, consisted of the STAI (Spielberger et al., 1983), the Eysenck Personality Questionnaire-revised (EPQ-R; Eysenck and Eysenck, 1991) and theBehavioral Inhibition/Behavioural Activation (BIS/BAS) scales (Carver and White, 1994). Only Extraversion, Neuroticism and BIS are reported here. Set 2, after the SST, consisted of 10 scales from the Personality Inventory for the DSM-5 (PID-5; American Psychiatric Association,, 2013). Only Depressiveness and Anxiousness are reported here.

EEG was recorded as in Shadli et al. (2021a). Briefly, the data were recorded with 32 channel Waveguard caps with Ag/AgCl electrodes, and an ASA Neurotechnology system (ANT Neuro, Enschede, The Netherlands) was used for recording with CPz as recording reference and electrode F8 of the International 10:20 system used for GCSR amplitude analysis. Ocular artefacts caused by eye blinks were detected via Fp1, and GND (anterior to Fz) was used as the ground electrode. All electrodes were later re-referenced to the common average of M1 and M2 mastoid electrodes. The impedance at each electrode was lowered to below 20 kΩ by injecting One Step Cleargel (H + H Medizinprodukte GbR, Münster, Germany) using a 10 mL syringe with a 16-gauge rounded needle (Precision Glide Needle, Becton Dickinson, Franklin lakes, NJ, USA) between the electrodes and the participant’s scalp. The sampling rate was 512 Hz, bandpass 1–36 Hz and down-sampled to 128 Hz for analysis.

### Procedure

All participants received the same general procedure, collection and processing to obtain GCSR. Participants provided written consent and then completed the first questionnaire set on a computer screen. They were connected to the EEG and undertook an SST described briefly below with full details as per the supplementary methods of Shadli et al. (2021a) available at https://doi.org/10.1038/s41598-021-99374-x. Participants then answered the second set of questionnaires. EEG caps were then removed, the gel cleaned off and they were thanked for their participation.

The SST was displayed on a computer screen, with verbal and screen instructions unchanged from Aron and Poldrack (2006). The task was modified by Shadli et al. (2015). GO trials involved the presentation of a white circle, which turned green when a left or right arrow appeared within it (GO stimulus) requiring a left or right mouse click, respectively. A smiley face appeared after a correct response and a frowny face after an incorrect response (e.g. opposite click response to arrow direction, or no click).

STOP trials were identical to the GO trials, except that a 1000 Hz auditory tone (STOP signal) played at a variable delay (SSD) after the GO stimulus appeared and participants had to inhibit all response mouse clicks. A smiley face followed successful response inhibition, and a frowny face followed any mouse click. Short and long stop signal delays (SSDs) were generated as a proportion of ongoing average GO reaction time as in Carter et al. (2003); medium SSDs were adjusted based on correct/incorrect responding to track 50% correct stopping. SSDs were thus separated into non-overlapping equal-sized short, medium and long groups.

In an initial block of testing (Block 0), 30 Go trials were presented without Stop trials. This was a primary choice reaction time (CRT) task similar to that used by Carter et al. (2003). No feedback about the participant’s GO reaction time was given during this phase. Otherwise, these trials were identical to GO trials presented later in the SST. The purpose of this testing was to allow adaptation to GO trial requirements and to record an initial Go mean reaction time (MRT). The Go MRT, in turn, was used to calculate starting SSD values and the GO reaction time that determined if feedback presentation was required to speed up responses.

The Stop task was like the primary CRT but with both Go and Stop trials. Participants were re-presented with the instructions at the start of each of the three blocks (Blocks 1–3). If a participant response occurred before the Go or Stop stimulus was presented, the trial was removed before data analysis.

The control of SSDs was identical to Shadli et al. (2015). A ‘staircase’ algorithm dictated SSD with short SSDs set to 20% of MRT for the prior 16 Go trials and long SSDs set to 80%. For the medium staircase, the delay for the first trial of a block was 45% of the previous 16 Go trial reaction times; and then changed depending on the most recent Stop trial performance. If participants successfully inhibited their response, SSD increased by 30 ms, if they were unsuccessful, SSD decreased by 30 ms.

The average Go reaction time at the end of the previous block was used to generate SSDs at the beginning of a block. Medium SSD was programmed so it never entered within 50 ms of other SSD staircases. This aimed to generate maximum go-stop conflict for the medium SSD while having a clear separation of the three SSD staircases (short, medium and long). We expected that the intermediate stair-casing system would track to 50% successful participant inhibitions, with approach and avoidance equal, maximum goal-conflict and BIS activation.

Successful matching was tested with IBM SPSS Statistics version 27 using ‘repeated measures’ ANOVA. The four groups (GAD-TR, GAD-not-TR, SAD-TR and SAD-not-TR) formed a 2 × 2 pair of between-subjects factors (diagnosis, GAD/SAD; resistance, TR/not-TR) and one repeated measures factor, demographics (age, EPQ score, STAI score, PID-Anx score and PID-dep score). An attempt was made to match TR-MDD and non-TR-GMD. However, due to low n available, these participants were not matched.

### Data analysis

EEG data were processed using a purpose-built Visual Basic program. Eyeblink artefacts were removed with a template fitting procedure (Zhang et al., 2017) and other artefacts by deletion of the epoch. For spectral analysis, a 1 s Hanning window was applied (centred on the 500 ms of the stop signal for both Stop and matching Go trials). This cosine wave extracts maximum power during the stop signal in stop trials and was applied to the Go trial adjacent to the stop trial only, omitting the other two Go trials of a four-trial set. A Fourier transform was then applied and converted to the power spectrum and a Log_10_ transform applied to normalise error variance. Spectra were averaged separately for Go and Stop trials, for each SSD type and block. Each participant had six averages for each block, 2 (stop, go) × 3 (short, medium long).

For each participant, GCSR values were calculated for each block of trials and frequency step extracted by the Fourier transform as per Shadli et al. (2021a). GCSR was computed as a nominal linear (Stop/Go) × quadratic (short/medium/long SSD) orthogonal polynomial contrast. Stopping-specific power was first extracted by subtracting the average Go from Stop power for each SSD type. To extract goal-conflict power, we subtracted the average of short and long SSD stop-specific power from medium SSD stop-specific power. Here, the greatest conflict-related power was expected to occur with the stop signal with a medium SSD, where going and stopping are equally likely. The lowest levels of conflict-related EEG power were expected during short and long SSD (~75% and ~25% correct stopping, respectively).

Previously, only the F8 channel was analysed for GCSR (associated with trait anxiety in SST for right-handed individuals). GCSR values were smoothed across frequency with a 3-pt running mean for direct comparison with Shadli et al. (2021a).

The GCSR frequency spectrum in the range 2–13 Hz (1–14 Hz before smoothing) in 1 Hz steps for F8 was analysed using repeated measures ANOVA in IBM SPSS (critical *p*-value: 0.05) and was carried out for blocks 2 and 3 averages for consistency with Shadli et al. (2021a). We extracted orthogonal polynomial components for the repeated measures frequency factor.

In one analysis, there were two between-subjects factors, each with two levels: diagnosis (GAD, SAD) and resistance (TR, non-TR), resulting from the four participants groups: GAD TR, GAD non-TR, SAD TR and SAD non-TR.

In a second analysis, TR-MDD and GMD participants were analysed separately from the anxiety groups with TR assessed as a single between-subjects factor. Because of the separate processing of these data from the anxiety groups, the GCSR frequency spectrum was in the range 2–10 Hz rather than 2–13 Hz. Using repeated measures ANOVA in IBM SPSS (critical *p*-value: 0.05), this was carried out for the average of blocks 2 and 3 and extracting polynomial components of frequency as in Shadli et al. (2021a). We also extracted orthogonal polynomial components for the repeated measures frequency factor.

## Results

### Demographics

**Figure 1.**
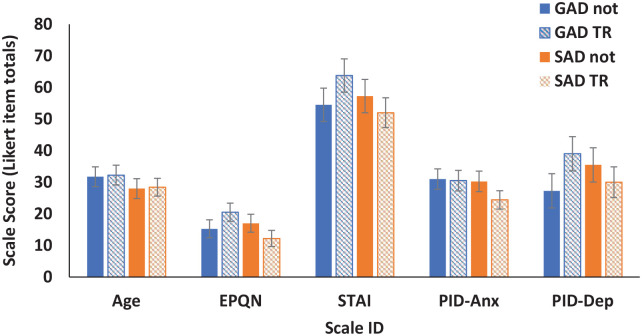
Matching demographics for GAD/SAD comparison. Means of age, Eysenck personality questionnaire neuroticism scale scores (EPQN; Eysenck and Eysenck, 1991), Spielberger state-trait anxiety inventory trait scale (STAI; Spielberger et al., 1983), personality inventory for the DSM-5 (American Psychiatric Association, 2013) anxiety (PID-Anx) and depression (PID-Dep) scores for treatment-responsive generalised anxiety disorder (GAD-not), treatment-resistant Generalised Anxiety Disorder (GAD-TR), treatment responsive Social Anxiety Disorder (SAD-not) and treatment-resistant Social Anxiety Disorder (SAD-TR). Error bars represent 2 standard errors.

Demographic data are shown in Figure 1 and Tables 1 and 2. Repeated measures ANOVA was carried out with diagnosis (GAD, SAD) and resistance (TR, not-TR) as between-subjects factors and demographics (age, EPQ, STAI, PID-Anx and PID-dep) as a repeated measures factor. Demographic matching was satisfactory across diagnosis and resistance. As demonstrated in Figure 1, there were substantial differences in demographic means consistent with the different scales used (demographics, *F* (1.969, 25.96) = 110.56, *p* < 0.0001, Greenhouse-Geisser corrected) but no interactions with diagnosis or resistance (all interactions *F* (1.969, 25.96) < 1.6, *p* > 0.24).

**Table 1.** Demographics for treatment-responsive generalised anxiety disorder (GAD-not), treatment-resistant generalised anxiety disorder (GAD-TR), treatment-responsive social anxiety disorder (SAD-not) and treatment-resistant social anxiety disorder (SAD-TR). Mean and standard error (SE) are given for age, Eysenck personality questionnaire neuroticism scale scores (EPQN; Eysenck and Eysenck, 1991), Spielberger state-trait anxiety inventory trait scale (STAI; Spielberger et al., 1983), personality inventory for the DSM-5 (American Psychiatric Association, 2013) anxiety (PID-Anx) and depression (PID-Dep). Gender *n* is given for males (M) and females (F).

	GAD-not		GAD-TR		SAD-not		SAD-TR	
Measure	Mean	SE	Mean	SE	Mean	SE	Mean	SE
Age	31.75	3.14	32.25	3.14	28.00	3.14	28.40	2.81
EPQN	15.25	2.85	20.50	2.85	17.00	2.85	12.20	2.55
STAI	54.50	5.28	63.75	5.29	57.25	5.29	52.00	4.73
PID-Anx	31.00	3.24	30.50	3.24	30.25	3.24	24.40	2.90
PID-Dep	27.25	5.43	39.00	5.43	35.50	5.43	30.00	4.85
M/F	1/3		1/3		1/4		1/4	

**Table 2.** Demographics for treatment-responsive comorbid generalised anxiety and depression (GMD-not) and treatment-resistant major depressive disorder (MDD-TR). Mean and standard error (SE) are given for age, Eysenck personality questionnaire neuroticism scale scores (EPQN; Eysenck and Eysenck, 1991), Spielberger state-trait anxiety inventory trait scale (STAI; Spielberger et al., 1983), personality inventory for the DSM-5 (American Psychiatric Association, 2013) anxiety (PID-Anx) and depression (PID-Dep). Gender *n* is given, gender *n* for males and females for abbreviations see, Figure 1.

	GMD-not		MDD-TR	
	Mean	SE	Mean	SE
Age	29.67	1.72	28.27	2.92
EPQN	15.13	1.28	16.64	1.08
STAI	56.40	1.93	61.91	2.26
PID-Anx	28.47	0.97	29.64	3.13
PID-Dep	31.07	1.95	39.55	3.13
M/F	2/13		6/5	

### GCSR theta variation with anxiety diagnosis and resistance

**Figure 2.**
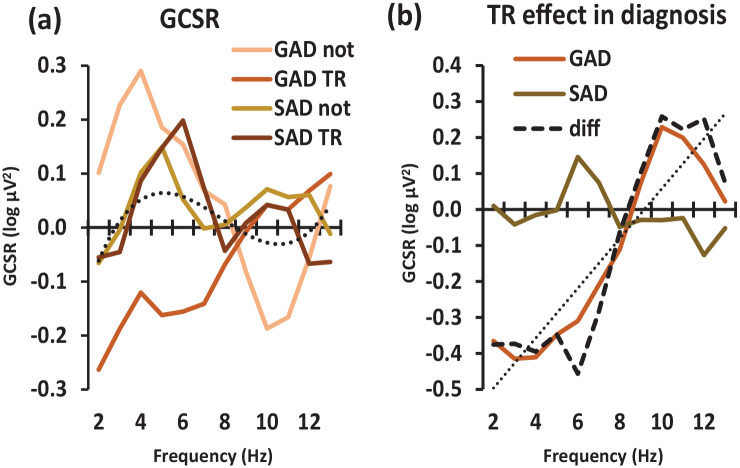
Variation in goal-conflict-specific electroencephalographic rhythmicity (GCSR) power with diagnosis and frequency across 2–13 Hz in anxiety groups. (a) Power variation in GAD-not-TR, GAD TR, SAD-not-TR and SAD TR patients. The dotted line shows the significant overall cubic trend when averaging across all diagnostic groups. (b) Effect of TR (i.e. the difference between TR and not GCSR scores) comparing GAD and SAD individuals. The dashed curve represents the TR × diagnosis × frequency interaction (i.e. the difference for TR/not across GAD/SAD) and the dotted straight line represents the marginal linear trend for this interaction. The departure of the dashed curve from the straight line at 6 and 10 Hz will result in the significant order 10 polynomial component of the interaction (see text). GAD: generalised anxiety disorder; SAD: social anxiety disorder; TR: treatment resistant.

Figure 2(a) shows GCSR strength plotted against frequency for GAD-TR, GAD-not-TR, SAD-TR and SAD-not-TR participants. *Change* in GCSR due to treatment resistance is shown for each diagnostic group in Figure 2(b).

Figure 2(a) shows a positive low-frequency GCSR (as expected from previous results) in the GAD-not-TR, SAD-not-TR and SAD TR groups but not the GAD-TR group. GAD-not-TR has a peak GCSR of 0.3 log µV^2^ at 4 Hz which drops to 0 log µV^2^ at 8 Hz. This then reduces to −0.2 log µV^2^ at 10 Hz and then returns to ~0.0 log µV^2^ at 13 Hz. By contrast, GAD-TR slowly increases from −0.3 to 0.0 log µV^2^ between 1 and 10 Hz with a maximum of 0.1 log µV^2^ between 10 and 11 Hz. SAD-not-TR peaks at 0.1 log µV^2^ at 5 Hz and 10–12 Hz but otherwise remains between −0.1 and 0.0 log µV^2^. Finally, SAD-TR increases from −0.1 log µV^2^ at 1 Hz to peak at 0.2 log µV^2^ at 6 Hz, falling to between 0.0 and 0.1 log µV^2^ at 7–10 Hz and then to −0.1 log µV^2^ at 10–11 Hz.

Across the groups, overall, power peaked in the region of 5 Hz with a zero or slightly below zero trough in the region of 10 Hz (dotted curve in Figure 2(a)). Repeated measures ANOVA extracted this as a significant cubic component (i.e. a ~ shaped curve with two inflections) of the overall effect of frequency (frequency (cubic), *F*(1, 14) = 5.63, *p* = 0.033). This pattern did not vary significantly across the groups, with no higher-order interactions of the cubic frequency component. Averaged over resistance, the diagnoses had somewhat different curve shapes with GAD peaking earlier than SAD (diagnosis × frequency (order 8) *F*(1, 14) = 5.79, *p* = 0.031).

Figure 2(b) shows that TR had little effect in the SAD group and abolished low-frequency GCSR (while perhaps increasing high frequency) in the GAD group. The resultant highest order interaction (the difference of the difference between the four treatment groups – dashed line in Figure 2(b)) appeared to have a linear trend from high to low frequency (diagnosis × resistance × frequency (lin), *F*(1, 14) = 4.32, *p* = 0.057, NS) with deviations from this linear trend at 6 Hz and 10 Hz (diagnosis × resistance × frequency (order 10), *F*(1, 14) = 7.15, *p* = 0.018). Note that, within ANOVA, these polynomial statistics are purely descriptive – they test for the presence of particular statistically independent (orthogonal) shapes within the tested space and do not fit supposed underlying polynomial functions that could be extrapolated outside the space. In this case, rather than detecting separate underlying linear and order 10 functions, their additive combination likely models simple power changes within a low (2–6 Hz) and a high (9–12 Hz) band – with the relatively abrupt shift between the bands in the region of 8 Hz generating a combination of low- and high-order polynomial components.

### GCSR theta variation with TR in depressed patients

**Figure 3.**
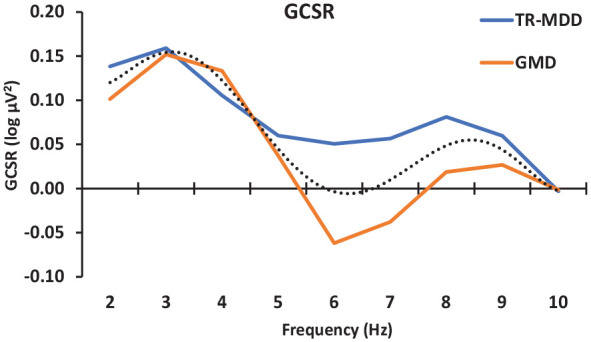
Variation goal-conflict-specific electroencephalographic rhythmicity (GCSR) power across 2–10 Hz in treatment-responsive comorbid generalised anxiety and depression (GMD) and treatment-resistant major depressive disorder (MDD-TR). The dotted line shows the significant overall order 4 trend generated by the presence of both low- and high-frequency band peaks. Note that, compared to the dotted line in Figure 2(a) (cubic, order 3), this has an extra inflection produced by the additional peak in the region of 8 Hz (with a trough at 6 Hz). The differences between the groups did not reach conventional levels of significance.

Figure 3 shows TR-MDD and GMD patients’ GCSR strength plotted against frequency. GCSR occurred at both low and high frequencies in TR-MDD and GMD, but the average across diagnoses tended to zero at 5–7 Hz. TR-MDD begins at 0.14 log µV^2^, peaks at 0.16 log µV^2^ at 3 Hz, drops to 0.11 log µV^2^ at 4 Hz, and then remains between 0.05 and 0.06 log µV^2^ at 5–7 Hz. At 8 Hz, it is 0.08 log µV^2^, at 9 Hz it is 0.06 log µV^2^ and at 10 Hz it is 0 log µV^2^ GMD begins at 0.1 log µV^2^ and peaks at 0.15 log µV^2^ at 3 Hz. It drops to 0.13 log µV^2^ at 4 Hz, then 0.04 log µV^2^ at 5 Hz, much like TR-MDD, but then drops to −0.06 log µV^2^ and −0.04 log µV^2^ at 6 and 7 Hz. It increases back to 0.03–0.00 log µV^2^ between 8 and 10 Hz.

Averaged across the two groups (Figure 3 dotted curve), GCSR steadily decreases across frequencies (frequency (lin), *F*(1, 24) = 5.683, *p* = 0.025), with clear peaks at low (3 Hz) and high (8–9 Hz) frequencies (frequency (order 4), *F* = 24.763, *p* < 0.0001). There may have been some higher-order variation from this common trend at intermediate frequencies; and the higher-order variation appears to have differed somewhat between the groups (frequency (order 6), *F* = 4.130, *p* = 0.053, NS; diagnosis × frequency (order 6), *F* = 3.899, *p* = 0.060, NS).

## Discussion

### Findings

We found significant positive low-frequency GCSR in all groups except for GAD-TR. TR had little effect on the GCSR of SAD or MDD patients (with, if anything, marginal increases). However, TR reversed low-frequency GCSR in GAD participants, while perhaps increasing it at higher frequencies. While the TR-MDD and GMD groups were not matched on demographics or diagnosis their low-frequency GCSR was very similar to each other and similar in magnitude to the anxiety groups (excluding TR-GAD) but perhaps at a lower frequency (3 Hz vs 4–6 Hz).

The results partially support our hypothesis that all anxiety participants (independent of TR) would demonstrate positive GCSR in the theta range. This was true for GAD-not-TR, GMD-not-TR (who have comorbid GAD), and both TR and not-TR SAD participants. However, completely contrary to the prediction, GAD-TR showed negative rather than positive GCSR in the low-frequency range. Also, apparently contrary to prediction, MDD-TR showed similar GCSR to the anxiety groups.

The failure of our prediction that GAD-TR would have particularly high GCSR is complete in that the reverse appears to be the case. To explain this, we should note that across a range of DSM diagnoses, all appear to include cases of elevated GCSR (though to differing extents), and none lack cases in the healthy GCSR range (Shadli et al., 2021a; see their Figure 4(e)). Thus, GCSR did not map to any single current diagnosis and is a biomarker for a process that may underlie a functional disorder that is, as yet, undefined.

A detailed discussion of the failure of current symptom-based diagnoses to map to expected syndromes, based on current neurology, is provided by McNaughton and Gray (2024), who also provide a detailed discussion of the neural basis of anxiolytic action. On this view, there would be at least two types of GAD, in the sense that it is currently diagnosed using symptom-based criteria.

The first type would be one where a generally hyperactive GCSR process contributes to the disorder (in combination with other traits; McNaughton and Glue, 2020) and so is sensitive to the selective drugs for anxiety, all of which reduce GCSR (McNaughton et al., 2013; Shadli et al., 2015). The most likely basis for this would be a hypersensitivity of the medial supramammillary area and an increase in hippocampal theta (and so prefrontal theta). That is, it would be the inverse of the changes in behaviour produced in this area by direct injection of anxiolytic drugs (Woodnorth and McNaughton, 2002). (Note that this same change could contribute to other current DSM diagnoses.)

The second type would be one where similar symptomatology arises, in the absence of positive GCSR, for some other reason. Given the lack of GCSR on which to act, the selective drugs for anxiety would be ineffective, contributing to TR. That is, no part of the theta input to the hippocampus would be hypersensitive (and so there is no excess frontal GCSR) and instead there is hypersensitivity of some target of hippocampal (or other downstream) output – most likely the Papez circuits to the prefrontal cortex (McNaughton and Vann, 2022). Note that changes in hippocampal theta and GCSR to drugs are immediate (McNaughton et al., 2007; McNaughton et al., 2013; Shadli et al., 2015), while therapeutic changes are slower, even for benzodiazepines (Wheatley, 1990). The hippocampal role in clinical (trait) anxiety, then, is likely to be mediated via memory-like processes in other structures, with the anxiolytics generating a form of anterograde amnesia (McNaughton and Gray, 2024).

### Interpretation

Inspection of the demographic data explains the unpredicted positive MDD results. The prediction was based on the assumption that the MDD group would have less comorbid anxiety than the GMD group (who were originally recruited as anxiety cases but subsequently diagnosed with comorbid depression). Table 2 shows that the MDD-TR group had numerically higher PID5-Anxiety and STAI-Trait scores than those of the GMD-not-TR group. The STAI-Trait difference is more than two standard errors but, given the similar difference in PID5-depression, this could be due to the presence of depression-related items in the STAI scale. The MDD-TR score for PID5-anxiety (29.6) is in the middle of the range for the anxiety groups with similar positive low-frequency GCSR power (24.4–31.0). GCSR, thus, appears to remain a biomarker for an anxiety process even when comorbid with depression. GCSR in depression that is not comorbid with anxiety remains to be tested.

We can apply related logic to the fact that SAD and GMD/MDD groups showed only marginal apparent increases in GCSR between TR and non-TR individuals. SAD and MDD show less sensitivity to selective drugs for anxiety than GAD (McNaughton and Glue, 2020: see their Table 1) and so their basic diagnosed dysfunction (especially in TR cases) would arise from a cause other than a hyperactive GCSR system. Their TR groups did not show elevated anxiety scores relative to non-TR nor relative to GAD-not-TR (which had both the highest PID5-anxiety score and the highest GCSR power peak). In all cases, then, positive GCSR would relate to anxiety symptoms but only in the case of GAD-not-TR would it reflect the causes as opposed to the consequences of the disorder.

The negative GCSR in GAD-TR individuals not only suggests that successful GAD treatment is linked to GCSR but also that this lack of the positive GCSR anxiety biomarker could, theoretically, be used as a TR biomarker in GAD populations. If the SST could be administered to individuals when they are diagnosed with GAD (via the current DSM or ICD frameworks), then their extracted (or lack thereof) GCSR could be used to predict the likelihood of these individuals responding to conventional treatments. If they demonstrate a strong positive GCSR (similar to GAD-non-TR in this study), this would indicate that these individuals would be responsive to conventional treatments (including SSRIs). However, if their GCSR is absent or very weak in the low-frequency range, this would indicate that these individuals do not have a GCSR that conventional drugs for anxiety can act on and thus there is a likelihood they will be resistant to conventional treatment and require, instead, treatments such as ketamine (Glue et al., 2017; Glue et al., 2018). At present, this remains only a theoretical possibility as GCSR in the SST is not sensitive or stable enough for individual testing.

### Limitations

These properties of the SST are also a limitation of the current study. GCSR measured using the SST has low test–retest reliability (Shadli et al., 2015), can be eliminated by prior relaxation (Shadli et al., 2019) and GCSR scores can differ widely between blocks (Shadli et al., 2015) with clinical effects being clearest only in the second two (Shadli et al., 2021a). We are currently exploring the simplification of a virtual-predator approach-escape conflict task (Fung et al., 2019; Qi et al., 2018) as an alternative to the SST and have preliminary evidence for test–retest reliability (Mia et al., 2024) and for drug validation in healthy students. We have produced but not fully tested a two-way avoidance version of this task (for an alternative two-way task, see Perkins et al., 2009). In addition to using a more stable paradigm, we have explored the use of an interpretable 3D convolutional neural network to improve signal extraction. This appears to produce a four-fold improvement in the prediction of anxious personality as measured by the STAI-T (Wang et al., 2019) but has not yet been validated with anxiolytic drugs.

A related limitation is that our anxiety sub-groups were small (*N* = 4–5), but, based on the drug data of Shadli et al. (2015), had about 0.85 power to detect meaningful differences at *p* < 0.05. That said, the sub-group difference clearly needs replication in larger, carefully matched, samples. Likewise, about 70% of participants were female. This is in line with New Zealand rates for GAD (65%) and MDD (63%) but less so for SAD (55%) as of 2006 (Oakley-Browne et al., 2006: see Table 3.1, p. 41) and possible sex differences need exploring in future work to determine generality. Note that for the GAD/SAD analyses gender was counterbalanced and so will not have been a confounding factor; whereas for the GMD/MDD analysis, gender is biased and could be acting as a confound.

Another limitation of this study and similar other studies is the variability of definitions of TR. Despite the prevalence of TR in anxious and depressive disorders, many different operationalisations exist with little to no consensus between clinicians and researchers (Bokma et al., 2019; Gaynes et al., 2020; Nierenberg and Amsterdam, 1990). Definitions of TR can range from the failure of any one treatment to ameliorate symptoms, a failure of two or more treatments and even specifically the failure of pharmacological or psychological treatments (or a combination of the two). The duration of treatment required for TR definitions can also vary between 1 and 6 months and some researchers even require increased anxiety severity over this time (Bokma et al., 2019). Such differences significantly reduce the generalisability of TR research in anxiety and depression (Bokma et al., 2019; Gaynes et al., 2020). Future studies could mitigate these issues by creating a clear definition that is universal over diagnoses and research studies. We chose to follow the suggestion from a systematic review of TR anxiety disorders that TR should be defined as ‘present after *both* at least one first-line pharmacological and one psychological treatment failure, provided for an adequate duration (at least 8 weeks) with anxiety severity remaining above a specified threshold’ (Bokma et al., 2019: 1, emphasis added). Given the problems that can arise with ineffective delivery of and ‘pseudo-resistance’ to medications (Roy-Byrne, 2015), we chose to also require a failure to at least two relevant medications.

An additional limitation is the unintentionally higher proportion of women than men (32 females, 12 males) recruited. As prior research has established structural and functional brain differences (Ritchie et al., 2018) and brain activity differences between males and females, this may have affected the GCSR observed. Studies have shown that female participants produce a higher amplitude of brain activity across many frequency bands than males (Kober et al., 2012; Wada et al., 1994). This is especially concerning when we consider that female participants seem to have a stronger frontal-midline theta than males (Kober et al., 2012) and females exhibit a positive relationship between frontal midline theta and dispositional anxiety that is not present in men (Osinsky et al., 2017). Although we measured the right frontal theta rather than the midline, these findings still suggest differential cortical activation in the frontal areas between the genders which may have confounded our results. A possible higher amplitude of activity in females than in males may have meant overall GCSR strength was higher than it would have been in an equally gender-split population. Future research in this area should ensure an even split between the genders to mitigate possible effects of gender on GCSR and use larger samples to allow estimation of gender effects.

A final limitation is that MDD participants were unmatched. This may have reduced some of the direct comparability between GMD (non-TR) and MDD (TR) participants. Some of the differences between GMD and MDD participants may have existed due to demographic factors such as age, questionnaire scores, handedness and gender. As possible differences in male vs female brain activation (Kober et al., 2012), brain activation with age (Ho et al., 2012), and left and right-handed GCSR (Shadli et al., 2021b) exist, this may have confounded the results. All of which may have been the reason for observed differences, or in this case lack thereof, between GMD and MDD. The fact that the non-TR participants in this example had a diagnosis (GMD) which is generally considered to be more severe than GAD or MDD alone (Coplan et al., 2015) and the fact that GMD generally has a higher GCSR than other diagnoses (Shadli et al., 2021a) may have also confounded these results. However, as discussed above, the measured anxiety and depression scale scores did not differ greatly between the groups. Future research would benefit from fully matching the MDD-TR and MDD-non-TR groups and also attempting to recruit groups of each with high and low anxiety comorbidity.

## Conclusions

The present study demonstrated positive GCSR in all diagnoses examined except GAD-TR. These findings are correlational but give support to the idea that successful GAD treatment is related to positive GCSR and that GAD-TR lacks GCSR that conventional drugs for anxiety could reduce. Future research is needed to determine if this is the case and to demonstrate the direction of any causality. SAD and MDD treatments, however, are likely related to dysfunctions other than excessive GCSR. A lack of the GCSR anxiety biomarker may be a biomarker for TR in GAD populations.
